# Kikuchi’s Disease Diagnosed by an Excisional Biopsy in a Patient With COVID-19

**DOI:** 10.7759/cureus.35251

**Published:** 2023-02-21

**Authors:** Aymen Al-Roubaie, Ali Uthuman, Thair Aldujaili, Khashayar Asadi, Majid Alabbood

**Affiliations:** 1 General Surgery, Goulburn Valley Health, Shepparton, AUS; 2 Internal Medicine, Goulburn Valley Health, Shepparton, AUS; 3 Pathology, Austin Health, Melbourne, AUS; 4 Internal Medicine, James Cook University, Mackay, AUS

**Keywords:** necrotising lymphadenopathy, excisional tissue biopsy, cervical lymphadenopathy, covid 19, s: kikuchi-fujimoto disease

## Abstract

COVID-19 is an ongoing pandemic caused by the novel coronavirus SARS-CoV-2. The clinical features of COVID-19 are myriad. Though it is a multisystem illness, it predominantly involves the respiratory system. There have been case reports on rare manifestations of COVID-19, of which COVID-19-related Kikuchi's disease is one of them. To our knowledge, this is the third reported case in the world.

We report a lady in her late 60s with COVID-19 infection and secondary bacterial pneumonia, which necessitated ICU admission, having ongoing fever spikes with high inflammatory markers and leukopenia. She was also found to have tender cervical lymphadenopathy on the third week of illness, whose biopsy revealed histiocytic necrotizing lymphadenitis in keeping with Kikuchi's disease. The patient had an uneventful recovery in two weeks without any intervention.

The pathophysiology of COVID-19-related Kikuchi's disease is unclear. However, COVID-19 is a viral illness that involves changes in interleukins. The latter is postulated in Kikuchi's disease.

## Introduction

Kikuchi’s disease, also known as histiocytic necrotizing lymphadenopathy, is extremely rare. There have been less than 50 reported cases worldwide. Kikuchi’s disease is of unknown etiology and was first reported in Japan in 1972. However, limited literature reports its occurrence post-viral infection [1].

It has a predilection to affect young women. Clinical presentation varies from mild fever, rash, lymphadenopathy, or muscle pain to headaches, nausea, vomiting, and malaise [2-6].

The diagnosis is challenging, and one of its clinical significance is that it macroscopically mimics malignant lymphoma or secondary deposit from solid tumors or skin malignancies and requires surgical excision for definite diagnosis at the cellular level. The treatment is usually supportive, with a self-limiting course in a few months [7,8].

We report a case of Kikuchi’s disease in a 68-year-old female with post-COVID-19 infection. To our knowledge, only one case of Kikuchi’s disease associated with COVID-19 has been reported in 2021 in a 32-year-old male [2].

## Case presentation

A 68-year-old female, triple vaccinated for COVID-19, presented to our emergency department with worsening dyspnoea for two days. She tested positive for COVID-19 PCR test four days earlier and was managed as moderate COVID-19 pneumonia with left lower zone secondary bacterial infection and discharged home on antibiotics. 

Five days later, she presented to the emergency department with ongoing fatigue, reduced oral intake, and dry cough. The patient also had intermittent fever. On initial examination, the findings suggested resolving left lower zone consolidation. Laboratory test results on serial days of admission are shown in Table 1.

**Table 1:** Laboratory investigations on serial days of admission

Laboratory test	Original Admission with COVID	Repeat Admission	First Week	Second Week	Third Week
Hb (115-155g/L)	92	92	81	83	86
WCC (10^9/L)	8.9	5.9	3.8	1.8	4.2
Platelets (10^9/L)	173	254	200	251	291
Creatinine (mmol/L)	140	103	98	102	138
eGFR	33	48	51	41	34
CRP (mg/L)	369	117	64	55	6
ESR (mm/h)			140		
Neutrophils				1.4	3.4
Lymphocytes				0.3	0.6
ANA				Positive Homogenous >640	
Anti-dsDNA				<10 IUmL	
ENA				Negative	
Rheumatoid factor				Negative	
Anti-CCP				<1.2 U/mL	
ANCA				Positive 3+P-ANCA MPO <1.0 IU/mL PR3 <0.6 IU/mL	
HIV				Negative	
Hepatitis B and C				Non-reactive	
EBV IgM				Negative	
CMV IgM				Negative	
LDH				280 U/L(120 - 250)	
Urine MCS	Negative	Negative	Negative	Negative	Negative
Blood Culture	Negative	Negative	Negative	Negative	Negative

**Figure 1:**
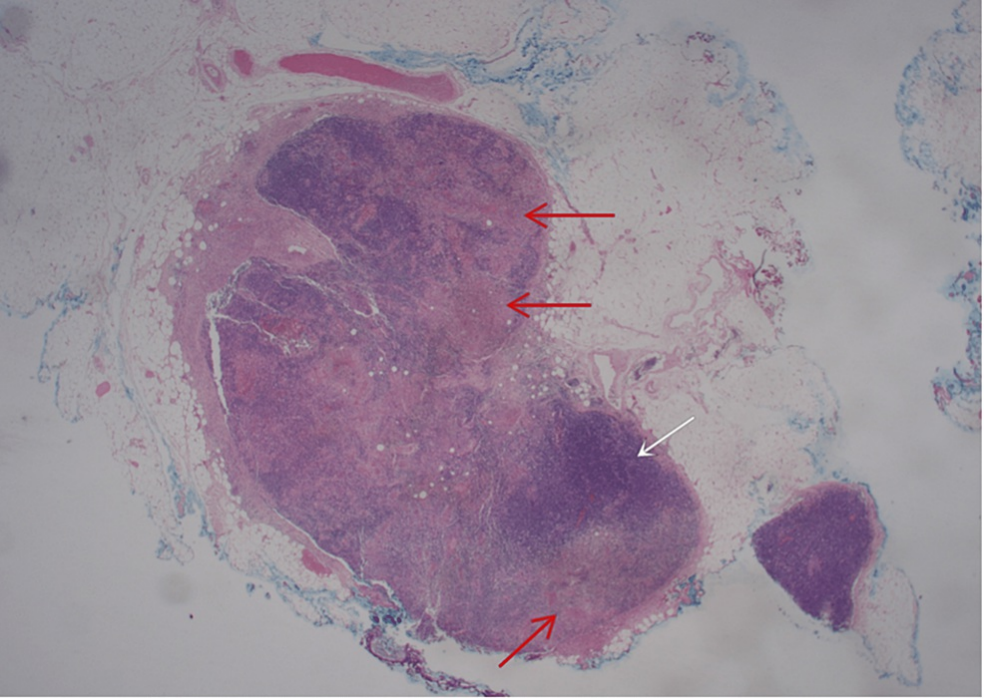
Red arrow necrosis, white arrow preserve nodal tissue

She was restarted on antibiotics to cover a possible nosocomial infection. However, she had ongoing fever with persistently elevated inflammatory markers and leukopenia. On the third week of hospital admission, she was noted to have tender cervical lymphadenopathy and underwent an excisional biopsy. The biopsy revealed necrotizing lymphadenitis, with irregular zones of geographic necrosis with karyorrhectic debris and a residual mixture of small polymorphous lymphocytes and histiocytes with peripheral areas of preserved paracortex with no associated suppuration, eosinophilia or granulomatous inflammation (Figure 1). 

**Figure 2:**
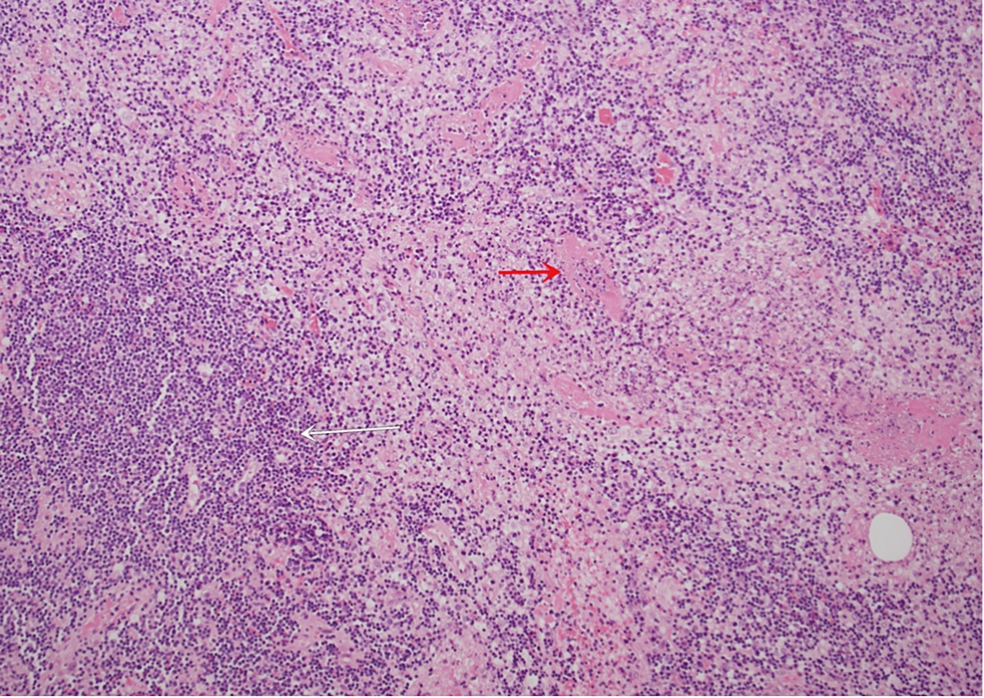
Fibrinoid necrosis of thin-walled blood vessels with vasculitis change

**Figure 3:**
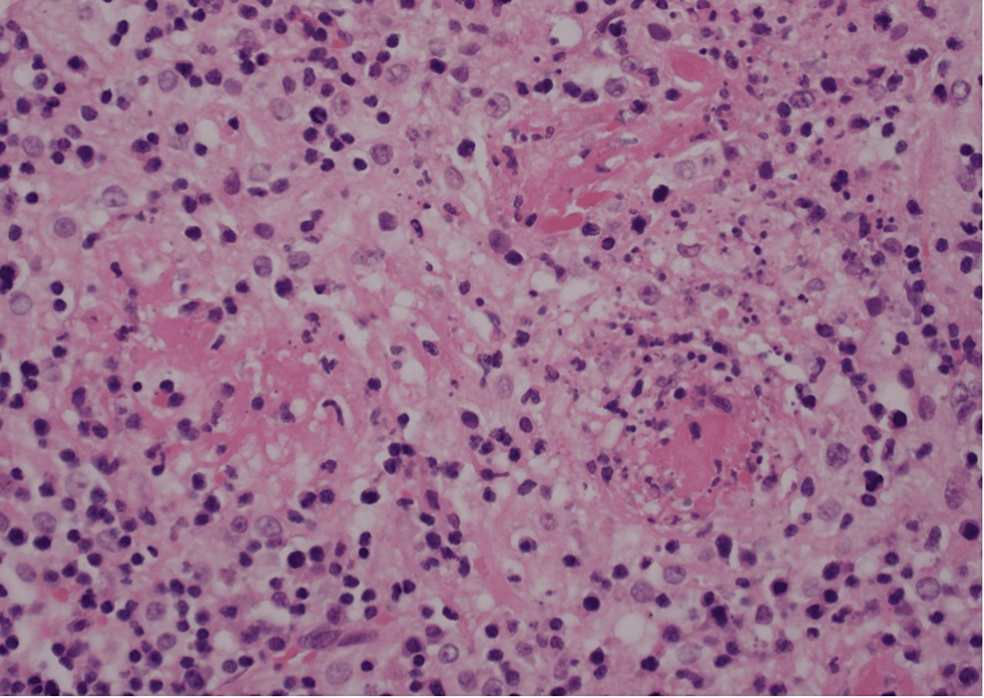
Fibrinoid necrosis of thin-walled blood vessels with vasculitis change and frequent microthrombi within and immediately adjacent to the zones of necrosis

**Figure 4:**
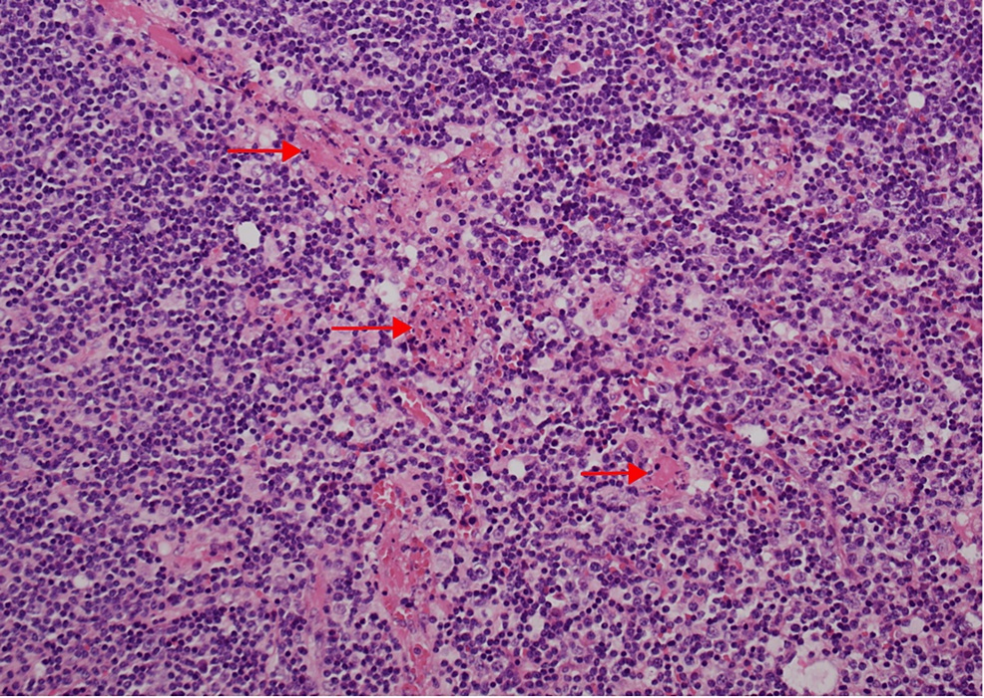
Frequent microthrombi within and immediately adjacent to the zones of necrosis

Furthermore, there was fibrinoid necrosis of thin-walled blood vessels with vasculitis change (Figures 2, 3) and frequent microthrombi within and immediately adjacent to the zones of necrosis (Figures 3, 4). 

Still, no vasculitis change or thrombi are seen in perinodal vessels. There was no evidence of metastatic malignancy, and there was no evidence of lymphoma. Flow cytometry studies on fresh tissue demonstrated three cell populations of lymphocytes, including normal mature T-cells (59% of lymphocytes) with a CD4:CD8 ratio of 1.36, polyclonal B-cells (40% of lymphocytes), and natural killer cells (1% of lymphocytes), with no abnormal lymphoid population. These findings support the diagnosis of Kikuchi's disease. 

Her past medical history is significant for type 2 diabetes mellitus, hypertension, hyperlipidaemia, hypothyroidism, chronic obstructive airway disease, and chronic kidney disease (stage IIIa).

After the initial course of antibiotics, she did not receive any antibiotics or further medications during the hospital workup. However, her fever spontaneously settled in the third week, and she was discharged. Subsequently, she was seen in the outpatient clinic twice, after two weeks and again after three months, and there were no features of lymphadenopathy. The patient clinically improved and was discharged from the clinic.

## Discussion

The Kikuchi-Fujimoto disease is an unusual self-limiting disease usually encountered in the Asian population. It has been described as related to autoimmune or infectious etiology [9]. The index case is a Caucasian woman who presented with suspicious cervical lymphadenopathy post-COVID-19 infection. A similar case was reported previously [2]. 

The Kikuchi-Fujimoto disease is a diagnostic dilemma and has a similar presentation to infectious or systemic lupus adenitis. The most important differential diagnosis of Kikuchi's disease is lymphoma and metastasis from skin malignancies. Therefore, accurate diagnosis is pivotal in its treatment. Surgical excision of the entire lymph node is necessary to obtain tissue diagnosis and rule out malignancies [3]. Kikuchi-Fujimoto disease is diagnosed histologically by distorted nodal architecture, asymmetrical paracortical areas of coagulative necrosis, and rich karyorrhectic debris [3]. 

Kikuchi-Fujimoto disease is treated conservatively with supportive treatments like analgesia and antipyretics. The disease itself usually runs a self-limiting course which has been observed in the index case.

## Conclusions

COVID-19 is a multisystemic disease that may affect any system and might have a variable presentation. Though respiratory symptoms are the most common manifestations, other system involvement is not uncommon. Lymph node enlargement needs careful consideration as it may be a manifestation of underlying multisystemic disease and needs to be differentiated from malignant or autoimmune etiology. Kikuchi's disease should be suspected in any patient with a recent COVID-19 infection and lymphadenopathy. Surgical excision is an important step in the workup of the suspicious case to exclude sinister causes and avoids treatment delay. Kikuchi's disease is a self-limiting disease that requires proper analgesia and supportive treatment only.
